# Supplementary material for: Patient-reported outcomes evaluation and assessment of facilitators and barriers to physical activity in the Transplantoux aerobic exercise intervention
Source: PLoS One. 2022 Oct 26;17(10):e0273497. doi: 10.1371/journal.pone.0273497 (PMC9605336; doi:10.1371/journal.pone.0273497)
Supplement: S1 Table — HCON: healthy participants; TxCON: control transplant recipients; TxCYC: transplant recipients participating in the cycling program; TxHIK: transplant recipients participating in the hiking program. (DOCX) [file pone.0273497.s002.docx]

**Supporting information:**

Table S1: Patient-reported outcomes for all groups on each timepoint

| Outcome | Time point | Parameter | 1. TxCYC | 2. TxHIK | 3. TxCON | 4. HCON |
| --- | --- | --- | --- | --- | --- | --- |
| Physical activity: MET-min per week | T1-0m | mean±SD | 5438±4717 | 5371±3946 | 4103±4073 | 4260±3597 |
|  | T2-3m | mean±SD | 6919±4679 | 6640±4007 | 4421±3866 | 5085±4165 |
|  | T3-6m | mean±SD | 6493±3815 | 5673±4813 | 4513±3899 | 5895±4562 |
|  | T4-9m | mean±SD | 6030±4420 | 4473±3732 | 4604±4558 | 4859±4103 |
|  | T5-12m | mean±SD | 5577±4555 | 6446±4279 | 4230±4422 | 4306±4012 |
| Physical activity: Status (1-3: low, moderate, high) | T1-0m | mean±SD | 2.62±0.57 | 2.67±0.49 | 2.25±0.67 | 2.51±0.66 |
|  | T2-3m | mean±SD | 2.78±0.52 | 2.78±0.43 | 2.40±0.65 | 2.61±0.59 |
|  | T3-6m | mean±SD | 2.68±0.62 | 2.53±0.62 | 2.31±0.72 | 2.59±0.61 |
|  | T4-9m | mean±SD | 2.44±0.74 | 2.47±0.62 | 2.34±0.65 | 2.56±0.56 |
|  | T5-12m | mean±SD | 2.51±0.66 | 2.77±0.44 | 2.29±0.68 | 2.33±0.69 |
| SF-36 physical component score | T1-0m | mean±SD | 80.39±14.08 | 80.73±9.45 | 62.29±21.41 | 83.90±13.77 |
|  | T2-3m | mean±SD | 82.13±11.61 | 82.05±11.88 | 63.89±20.30 | 83.93±14.34 |
|  | T3-6m | mean±SD | 86.85±11.36 | 83.61±11.02 | 66.39±21.46 | 89.53±13.08 |
|  | T4-9m | mean±SD | 85.64±10.28 | 81.90±12.55 | 67.07±21.32 | 89.49±11.58 |
|  | T5-12m | mean±SD | 85.92±12.04 | 83.61±10.89 | 66.65±21.32 | 84.65±16.46 |
| SF-36 mental component score | T1-0m | mean±SD | 83.09±17.19 | 83.30±13.10 | 73.42±19.82 | 82.83±12.30 |
|  | T2-3m | mean±SD | 83.14±17.14 | 85.76±12.13 | 73.75±20.80 | 85.15±9.52 |
|  | T3-6m | mean±SD | 85.76±15.16 | 86.73±9.72 | 74.72±20.55 | 88.68±10.39 |
|  | T4-9m | mean±SD | 88.12±10.91 | 87.19±5.59 | 75.07±20.31 | 85.76±12.72 |
|  | T5-12m | mean±SD | 87.59±10.64 | 86.40±9.75 | 73.71±20.73 | 83.24±14.79 |
| EuroQol VAS | T1-0m | mean±SD | 81.49±11.65 | 81.72±7.08 | 68.62±18.36 | 80.51±14.77 |
|  | T2-3m | mean±SD | 81.89±10.89 | 81.94±9.08 | 71.37±16.75 | 83.65±11.70 |
|  | T3-6m | mean±SD | 84.40±11.12 | 82.18±9.59 | 71.67±17.89 | 84.82±11.74 |
|  | T4-9m | mean±SD | 82.32±10.32 | 82.47±11.14 | 71.19±18.39 | 84.32±11.69 |
|  | T5-12m | mean±SD | 82.51±11.03 | 85.69±9.30 | 68.97±19.74 | 82.38±12.80 |
| Mental health | T1-0m | mean±SD | 1.19±2.74 | 0.50±0.92 | 1.86±2.84 | 1.29±2.41 |
|  | T2-3m | mean±SD | 0.71±1.79 | 0.33±0.84 | 1.74±2.77 | 0.41±1.10 |
|  | T3-6m | mean±SD | 1.00±2.22 | 0.24±0.56 | 1.44±2.57 | 0.34±1.11 |
|  | T4-9m | mean±SD | 0.59±1.48 | 0.53±1.50 | 1.52±2.72 | 0.81±2.07 |
|  | T5-12m | mean±SD | 0.51±1.15 | 0.23±0.44 | 1.95±3.14 | 1.04±1.92 |
| Depressive symptoms | T1-0m | mean±SD | 3.40±6.42 | 2.78±3.15 | 6.19±7.22 | 3.08±4.65 |
|  | T2-3m | mean±SD | 3.29±5.56 | 1.78±3.14 | 6.16±7.61 | 1.87±3.01 |
|  | T3-6m | mean±SD | 3.65±7.97 | 1.41±2.32 | 5.43±7.15 | 1.17±2.52 |
|  | T4-9m | mean±SD | 2.78±4.90 | 2.82±4.59 | 5.62±7.53 | 2.58±4.53 |
|  | T5-12m | mean±SD | 2.97±6.01 | 2.31±3.99 | 6.46±8.11 | 2.08±2.70 |
| Anxiety | T1-0m | mean±SD | 2.51±3.35 | 5.11±4.81 | 5.59±5.83 | 2.35±3.87 |
|  | T2-3m | mean±SD | 1.87±3.23 | 3.56±2.62 | 5.76±6.54 | 1.37±2.28 |
|  | T3-6m | mean±SD | 2.95±7.50 | 2.59±2.72 | 5.22±5.85 | 0.62±1.32 |
|  | T4-9m | mean±SD | 2.05±2.85 | 3.53±5.64 | 5.27±6.24 | 2.00±3.05 |
|  | T5-12m | mean±SD | 1.26±1.62 | 3.23±2.24 | 5.49±6.02 | 1.75±3.16 |
| Stress | T1-0m | mean±SD | 5.91±6.65 | 7.89±5.84 | 8.60±7.65 | 6.59±6.19 |
|  | T2-3m | mean±SD | 5.29±5.45 | 6.11±7.18 | 8.92±8.24 | 5.19±4.56 |
|  | T3-6m | mean±SD | 5.35±8.44 | 4.47±6.10 | 8.26±7.49 | 2.80±4.15 |
|  | T4-9m | mean±SD | 4.34±5.48 | 6.59±5.69 | 8.22±7.61 | 5.42±5.90 |
|  | T5-12m | mean±SD | 4.17±4.38 | 6.46±4.98 | 8.88±7.67 | 4.92±5.04 |

HCON: healthy participants; TxCON: control transplant recipients; TxCYC: transplant recipients participating in the cycling program; TxHIK: transplant recipients participating in the hiking program.
