# Supplementary material for: Patient-reported outcomes evaluation and assessment of facilitators and barriers to physical activity in the Transplantoux aerobic exercise intervention
Source: PLoS One. 2022 Oct 26;17(10):e0273497. doi: 10.1371/journal.pone.0273497 (PMC9605336; doi:10.1371/journal.pone.0273497)
Supplement: S2 Table — * 1: cycling transplant recipients; 2: hiking transplant recipients; 3: control transplant recipients; 4: healthy participants. (DOCX) [file pone.0273497.s003.docx]

**Supporting information:**

Table S2: Between-group differences of patient-reported outcomes at baseline (T1)

| General linear models | Contrast * | Estimate | SE | Lower | Upper | DF | t-Value | Pr > \|t\| | Odds ratio |
| --- | --- | --- | --- | --- | --- | --- | --- | --- | --- |
| Physical activity: MET-min per week (square root) | 1vs2 | -0.7270 | 8.0418 | -16.5450 | 15.0911 | 340 | -0.09 | 0.9280 | / |
|  | 1vs3 | 11.1853 | 4.7413 | 1.8594 | 20.5112 | 340 | 2.36 | 0.0189 | / |
|  | 1vs4 | 7.5420 | 5.2945 | -2.8721 | 17.9562 | 340 | 1.42 | 0.1552 | / |
|  | 2vs3 | 11.9123 | 7.1255 | -2.1034 | 25.9280 | 340 | 1.67 | 0.0955 | / |
|  | 2vs4 | 8.2690 | 7.5051 | -6.4932 | 23.0312 | 340 | 1.10 | 0.2713 | / |
|  | 3vs4 | -3.6433 | 3.7595 | -11.0382 | 3.7516 | 340 | -0.97 | 0.3332 | / |
| Physical activity: status (1-3: low, moderate, high) | 1vs2 | -0.0899 | 0.5769 | -1.2243 | 1.0445 | 364 | -0.16 | 0.8763 | 0.91 (0.29-2.84) |
|  | 1vs3 | 1.1269 | 0.3324 | 0.4733 | 1.7806 | 364 | 3.39 | 0.0008 | 3.09 (1.61-5.93) |
|  | 1vs4 | 0.3326 | 0.3702 | -0.3955 | 1.0606 | 364 | 0.90 | 0.3696 | 1.39 (0.67-2.89) |
|  | 2vs3 | 1.2168 | 0.5083 | 0.2173 | 2.2164 | 364 | 2.39 | 0.0172 | 3.38 (1.24-9.17) |
|  | 2vs4 | 0.4225 | 0.5337 | -0.6271 | 1.4721 | 364 | 0.79 | 0.4291 | 1.53 (0.53-4.36) |
|  | 3vs4 | -0.7944 | 0.2493 | -1.2846 | -0.3041 | 364 | -3.19 | 0.0016 | 0.45 (0.27-0.74) |
| SF-36 physical component score (squared) | 1vs2 | 55.9912 | 642.09 | -1206.66 | 1318.64 | 365 | 0.09 | 0.9306 | / |
|  | 1vs3 | 2320.33 | 373.31 | 1586.22 | 3054.43 | 365 | 6.22 | <.0001 | / |
|  | 1vs4 | -570.49 | 416.09 | -1388.73 | 247.76 | 365 | -1.37 | 0.1712 | / |
|  | 2vs3 | 2264.33 | 568.59 | 1146.20 | 3382.47 | 365 | 3.98 | <.0001 | / |
|  | 2vs4 | -626.48 | 597.56 | -1801.56 | 548.61 | 365 | -1.05 | 0.2951 | / |
|  | 3vs4 | -2890.81 | 290.10 | -3461.29 | -2320.34 | 365 | -9.96 | <.0001 | / |
| SF-36 mental component score (squared) | 1vs2 | 91.2221 | 647.64 | -1182.40 | 1364.84 | 362 | 0.14 | 0.8881 | / |
|  | 1vs3 | 1411.46 | 377.03 | 670.02 | 2152.90 | 362 | 3.74 | 0.0002 | / |
|  | 1vs4 | 182.27 | 419.70 | -643.08 | 1007.62 | 362 | 0.43 | 0.6643 | / |
|  | 2vs3 | 1320.24 | 573.83 | 191.77 | 2448.71 | 362 | 2.30 | 0.0220 | / |
|  | 2vs4 | 91.0500 | 602.73 | -1094.24 | 1276.34 | 362 | 0.15 | 0.8800 | / |
|  | 3vs4 | -1229.19 | 293.24 | -1805.85 | -652.53 | 362 | -4.19 | <.0001 | / |
| EuroQol VAS (squared) | 1vs2 | 47.4858 | 580.83 | -1094.70 | 1189.67 | 365 | 0.08 | 0.9349 | / |
|  | 1vs3 | 1729.18 | 337.69 | 1065.11 | 2393.25 | 365 | 5.12 | <.0001 | / |
|  | 1vs4 | 76.5279 | 376.40 | -663.65 | 816.71 | 365 | 0.20 | 0.8390 | / |
|  | 2vs3 | 1681.69 | 514.35 | 670.24 | 2693.15 | 365 | 3.27 | 0.0012 | / |
|  | 2vs4 | 29.0421 | 540.55 | -1033.93 | 1092.02 | 365 | 0.05 | 0.9572 | / |
|  | 3vs4 | -1652.65 | 262.42 | -2168.70 | -1136.60 | 365 | -6.30 | <.0001 | / |
| Mental health | 1vs2 | 0.1611 | 0.6037 | -1.0262 | 1.3485 | 354 | 0.27 | 0.7897 | 1.17 (0.36–3.85) |
|  | 1vs3 | -0.8102 | 0.3477 | -1.4940 | -0.1265 | 354 | -2.33 | 0.0203 | 0.44 (0.22–0.88) |
|  | 1vs4 | -0.3113 | 0.3869 | -1.0722 | 0.4496 | 354 | -0.80 | 0.4216 | 0.73 (0.34–1.57) |
|  | 2vs3 | -0.9714 | 0.5266 | -2.0070 | 0.0643 | 354 | -1.84 | 0.0659 | 0.38 (0.13–1.07) |
|  | 2vs4 | -0.4724 | 0.5533 | -1.5605 | 0.6157 | 354 | -0.85 | 0.3938 | 0.62 (0.21–1.85) |
|  | 3vs4 | 0.4989 | 0.2494 | 0.00841 | 0.9895 | 354 | 2.00 | 0.0462 | 1.65 (1.01–2.69) |
| Depressive symptoms | 1vs2 | -0.4085 | 0.5101 | -1.4117 | 0.5946 | 363 | -0.80 | 0.4237 | 0.66 (0.24–1.81) |
|  | 1vs3 | -1.1140 | 0.3177 | -1.7387 | -0.4892 | 363 | -3.51 | 0.0005 | 0.33 (0.18–0.61) |
|  | 1vs4 | -0.2949 | 0.3489 | -0.9811 | 0.3912 | 363 | -0.85 | 0.3985 | 0.74 (0.37–1.48) |
|  | 2vs3 | -0.7054 | 0.4378 | -1.5664 | 0.1555 | 363 | -1.61 | 0.1080 | 0.49 (0.21–1.17) |
|  | 2vs4 | 0.1136 | 0.4620 | -0.7950 | 1.0222 | 363 | 0.25 | 0.8059 | 1.12 (0.45–2.78) |
|  | 3vs4 | 0.8190 | 0.2319 | 0.3630 | 1.2751 | 363 | 3.53 | 0.0005 | 2.27 (1.44–3.58) |
| Anxiety | 1vs2 | -0.8220 | 0.4896 | -1.7847 | 0.1408 | 362 | -1.68 | 0.0940 | 0.44 (0.17–1.15) |
|  | 1vs3 | -0.9894 | 0.2791 | -1.5383 | -0.4405 | 362 | -3.54 | 0.0004 | 0.37 (0.22–0.64) |
|  | 1vs4 | 0.3907 | 0.3145 | -0.2277 | 1.0091 | 362 | 1.24 | 0.2149 | 1.48 (0.80–2.74) |
|  | 2vs3 | -0.1675 | 0.4386 | -1.0301 | 0.6951 | 362 | -0.38 | 0.7028 | 0.85 (0.36–2.00) |
|  | 2vs4 | 1.2127 | 0.4673 | 0.2938 | 2.1316 | 362 | 2.60 | 0.0098 | 3.36 (1.34–8.43) |
|  | 3vs4 | 1.3802 | 0.2380 | 0.9121 | 1.8482 | 362 | 5.80 | <.0001 | 3.98 (2.49–6.35) |
| Stress | 1vs2 | -0.7852 | 0.4864 | -1.7417 | 0.1714 | 361 | -1.61 | 0.1074 | 0.46 (0.18–1.19) |
|  | 1vs3 | -0.7042 | 0.2813 | -1.2575 | -0.1509 | 361 | -2.50 | 0.0128 | 0.50 (0.28–0.86) |
|  | 1vs4 | -0.3060 | 0.3102 | -0.9161 | 0.3040 | 361 | -0.99 | 0.3245 | 0.74 (0.40–1.36) |
|  | 2vs3 | 0.0809 | 0.4331 | -0.7707 | 0.9326 | 361 | 0.19 | 0.8518 | 1.08 (0.46–2.54) |
|  | 2vs4 | 0.4791 | 0.4535 | -0.4127 | 1.3709 | 361 | 1.06 | 0.2914 | 1.61 (0.66–3.94) |
|  | 3vs4 | 0.3982 | 0.2195 | -0.0334 | 0.8298 | 361 | 1.81 | 0.0705 | 1.49 (0.97–2.29) |

* 1: cycling transplant recipients; 2: hiking transplant recipients; 3: control transplant recipients; 4: healthy participants.
