# Supplementary material for: Patient-reported outcomes evaluation and assessment of facilitators and barriers to physical activity in the Transplantoux aerobic exercise intervention
Source: PLoS One. 2022 Oct 26;17(10):e0273497. doi: 10.1371/journal.pone.0273497 (PMC9605336; doi:10.1371/journal.pone.0273497)
Supplement: S3 Table — * 1: cycling transplant recipients; 2: hiking transplant recipients; 3: control transplant recipients; 4: healthy participants. (DOCX) [file pone.0273497.s004.docx]

**Supporting information:**

Table S3. Regression slopes per study group and slope contrasts of patient-reported outcomes over time between baseline (T1) and intervention (T3, month 6)

| General mixed models | |  |  |  |  |  |  |  |  |
| --- | --- | --- | --- | --- | --- | --- | --- | --- | --- |
| Variable | Group | Estimate | SE | DF | t Value | Pr > \|t\| | Lower | Upper | OR (95%CI) |
| Physical activity: MET-min per week (square root) | 1 TxCYC | 2.6181 | 1.2542 | 568 | 2.09 | 0.0373 | 0.1547 | 5.0815 | / |
|  | 2 TxHIK | 0.07759 | 1.9535 | 568 | 0.04 | 0.9683 | –3.7593 | 3.9145 | / |
|  | 3 TxCON | 1.9891 | 0.6260 | 568 | 3.18 | 0.0016 | 0.7596 | 3.2186 | / |
|  | 4 HCON | 3.3946 | 1.0019 | 568 | 3.39 | 0.0008 | 1.4267 | 5.3625 | / |
|  | 1 vs 2 | 2.5405 | 2.3214 | 568 | 1.09 | 0.2743 | –2.0191 | 7.1002 | / |
|  | 1 vs 3 | 0.6290 | 1.4017 | 568 | 0.45 | 0.6538 | –2.1242 | 3.3822 | / |
|  | 2 vs 3 | -1.9115 | 2.0513 | 568 | -0.93 | 0.3518 | –5.9406 | 2.1175 | / |
|  | 1 vs 4 | -0.7765 | 1.6052 | 568 | -0.48 | 0.6288 | –3.9294 | 2.3765 | / |
|  | 2 vs 4 | -3.3170 | 2.1954 | 568 | -1.51 | 0.1314 | –7.6291 | 0.9951 | / |
| SF-36 physical component score (squared) | 1 TxCYC | 331.66 | 85.3001 | 620 | 3.89 | 0.0001 | 164.15 | 499.17 | / |
|  | 2 TxHIK | 194.22 | 131.74 | 620 | 1.47 | 0.1409 | –64.479 | 452.93 | / |
|  | 3 TxCON | 189.07 | 41.1487 | 620 | 4.59 | <.0001 | 108.26 | 269.87 | / |
|  | 4 HCON | 269.50 | 66.1511 | 620 | 4.07 | <.0001 | 139.59 | 399.41 | / |
|  | 1 vs 2 | 137.43 | 156.94 | 620 | 0.88 | 0.3815 | –170.77 | 445.64 | / |
|  | 1 vs 3 | 142.59 | 94.7065 | 620 | 1.51 | 0.1327 | –43.3912 | 328.58 | / |
|  | 2 vs 3 | 5.1588 | 138.01 | 620 | 0.04 | 0.9702 | –265.87 | 276.19 | / |
|  | 1 vs 4 | 62.1569 | 107.94 | 620 | 0.58 | 0.5649 | –149.82 | 274.14 | / |
|  | 2 vs 4 | -75.2774 | 147.41 | 620 | -0.51 | 0.6098 | –364.77 | 214.21 | / |
| SF-36 mental component score (squared) | 1 TxCYC | 61.2174 | 88.1546 | 617 | 0.69 | 0.4877 | –111.90 | 234.34 | / |
|  | 2 TxHIK | 142.07 | 136.16 | 617 | 1.04 | 0.2972 | –125.32 | 409.47 | / |
|  | 3 TxCON | 76.6015 | 42.7207 | 617 | 1.79 | 0.0735 | –7.2942 | 160.50 | / |
|  | 4 HCON | 303.64 | 68.3528 | 617 | 4.44 | <.0001 | 169.41 | 437.87 | / |
|  | 1 vs 2 | -80.8564 | 162.21 | 617 | -0.50 | 0.6183 | –399.40 | 237.69 | / |
|  | 1 vs 3 | -15.3840 | 97.9607 | 617 | -0.16 | 0.8753 | –207.76 | 176.99 | / |
|  | 2 vs 3 | 65.4723 | 142.71 | 617 | 0.46 | 0.6465 | –214.78 | 345.72 | / |
|  | 1 vs 4 | -242.42 | 111.55 | 617 | -2.17 | 0.0301 | –461.48 | -23.358 | / |
|  | 2 vs 4 | -161.56 | 152.36 | 617 | -1.06 | 0.2894 | –460.76 | 137.63 | / |
| EuroQol VAS (squared) | 1 TxCYC | 133.30 | 82.5712 | 621 | 1.61 | 0.1070 | –28.8522 | 295.45 | / |
|  | 2 TxHIK | 51.6361 | 127.59 | 621 | 0.40 | 0.6858 | –198.92 | 302.20 | / |
|  | 3 TxCON | 112.55 | 39.7200 | 621 | 2.83 | 0.0048 | 34.5453 | 190.55 | / |
|  | 4 HCON | 148.17 | 63.9854 | 621 | 2.32 | 0.0209 | 22.5123 | 273.82 | / |
|  | 1 vs 2 | 81.6642 | 151.98 | 621 | 0.54 | 0.5912 | –216.79 | 380.12 | / |
|  | 1 vs 3 | 20.7533 | 91.6279 | 621 | 0.23 | 0.8209 | –159.18 | 200.69 | / |
|  | 2 vs 3 | -60.9110 | 133.63 | 621 | -0.46 | 0.6487 | –323.33 | 201.51 | / |
|  | 1 vs 4 | -14.8659 | 104.46 | 621 | -0.14 | 0.8869 | –220.01 | 190.27 | / |
|  | 2 vs 4 | -96.5301 | 142.74 | 621 | -0.68 | 0.4991 | –376.83 | 183.77 | / |
| Generalized mixed models | |  |  |  |  |  |  |  |  |
| Variable | Group | Estimate | SE | DF | t Value | Pr > \|t\| | Lower | Upper | OR (95%CI) |
| Physical activity: status | 1 TxCYC | 0.1370 | 0.1951 | 617 | 0.70 | 0.4829 | -0.2462 | 0.5202 | 1.15 (0.78-1.68) |
|  | 2 TxHIK | -0.2998 | 0.2793 | 617 | -1.07 | 0.2834 | -0.8483 | 0.2486 | 0.74 (0.42-1.28) |
|  | 3 TxCON | 0.0775 | 0.0760 | 617 | 1.02 | 0.3084 | -0.0718 | 0.2268 | 1.08 (0.93-1.25) |
|  | 4 HCON | 0.1246 | 0.1365 | 617 | 0.91 | 0.3616 | -0.1434 | 0.3927 | 1.13 (0.87-1.48) |
|  | 1 vs 2 | 0.4368 | 0.3408 | 617 | 1.28 | 0.2004 | -0.2325 | 1.1062 | 1.55 (0.79-3.02) |
|  | 1 vs 3 | 0.0595 | 0.2093 | 617 | 0.28 | 0.7764 | -0.3516 | 0.4706 | 1.06 (0.70-1.60) |
|  | 2 vs 3 | -0.3774 | 0.2896 | 617 | -1.30 | 0.1930 | -0.9461 | 0.1914 | 0.69 (0.39-1.21) |
|  | 1 vs 4 | 0.0124 | 0.2381 | 617 | 0.05 | 0.9586 | -0.4551 | 0.4799 | 1.01 (0.63-1.61) |
|  | 2 vs 4 | -0.4245 | 0.3110 | 617 | -1.36 | 0.1728 | -1.0352 | 0.1863 | 0.65 (0.35-1.20) |
| Mental health | 1 TxCYC | 0.05223 | 0.1866 | 610 | 0.28 | 0.7796 | –0.3142 | 0.4186 | 1.05 (0.73–1.52) |
|  | 2 TxHIK | -0.2103 | 0.3217 | 610 | -0.65 | 0.5135 | –0.8422 | 0.4215 | 0.81 (0.43–1.52) |
|  | 3 TxCON | -0.1747 | 0.07609 | 610 | -2.30 | 0.0220 | –0.3241 | -0.02526 | 0.84 (0.72–0.98) |
|  | 4 HCON | -0.5944 | 0.1683 | 610 | -3.53 | 0.0004 | –0.9248 | -0.2639 | 0.55 (0.40–0.77) |
|  | 1 vs 2 | 0.2626 | 0.3719 | 610 | 0.71 | 0.4805 | –0.4679 | 0.9930 | 1.30 (0.63–2.70) |
|  | 1 vs 3 | 0.2269 | 0.2016 | 610 | 1.13 | 0.2609 | –0.1691 | 0.6229 | 1.25 (0.85–1.86) |
|  | 2 vs 3 | -0.03565 | 0.3305 | 610 | -0.11 | 0.9141 | –0.6847 | 0.6134 | 0.97 (0.50–1.85) |
|  | 1 vs 4 | 0.6466 | 0.2517 | 610 | 2.57 | 0.0104 | 0.1524 | 1.1408 | 1.91 (1.16–3.13) |
|  | 2 vs 4 | 0.3840 | 0.3628 | 610 | 1.06 | 0.2902 | –0.3284 | 1.0964 | 1.47 (0.72–2.99) |
| Stress | 1 TxCYC | -0.2853 | 0.1477 | 617 | -1.93 | 0.0539 | –0.5754 | 0.00476 | 0.75 (0.56–1.00) |
|  | 2 TxHIK | -0.6444 | 0.2279 | 617 | -2.83 | 0.0048 | –1.0920 | -0.1968 | 0.53 (0.34–0.82) |
|  | 3 TxCON | -0.04125 | 0.06896 | 617 | -0.60 | 0.5500 | –0.1767 | 0.09417 | 0.96 (0.94–1.10) |
|  | 4 HCON | -0.8234 | 0.1252 | 617 | -6.57 | <.0001 | –1.0693 | -0.5774 | 0.44 (0.34–0.56) |
|  | 1 vs 2 | 0.3591 | 0.2710 | 617 | 1.33 | 0.1856 | 0.05 | -0.1731 | 1.43 (0.84-2.44) |
|  | 1 vs 3 | -0.2441 | 0.1629 | 617 | -1.50 | 0.1346 | –0.5641 | 0.07590 | 0.78 (0.57–1.08) |
|  | 2 vs 3 | -0.6032 | 0.2380 | 617 | -2.53 | 0.0115 | –1.0705 | -0.1358 | 0.55 (0.34–0.87) |
|  | 1 vs 4 | 0.5380 | 0.1923 | 617 | 2.80 | 0.0053 | 0.1604 | 0.9157 | 1.71 (1.17–2.50) |
|  | 2 vs 4 | 0.1790 | 0.2581 | 617 | 0.69 | 0.4883 | –0.3280 | 0.6859 | 1.20 (0.72–1.99) |
| Depressive symptoms | 1 TxCYC | 0.07750 | 0.1745 | 619 | 0.44 | 0.6571 | –0.2652 | 0.4202 | 1.08 (0.77–1.52) |
|  | 2 TxHIK | -0.4782 | 0.2676 | 619 | -1.79 | 0.0744 | –1.0038 | 0.04735 | 0.62 (0.67–1.05) |
|  | 3 TxCON | -0.1349 | 0.07670 | 619 | -1.76 | 0.0791 | –0.2855 | 0.01573 | 0.87 (0.75–1.02) |
|  | 4 HCON | -0.6462 | 0.1417 | 619 | -4.56 | <.0001 | –0.9245 | -0.3680 | 0.52 (0.40–0.69) |
|  | 1 vs 2 | 0.5557 | 0.3199 | 619 | 1.74 | 0.0828 | –0.0724 | 1.1839 | 1.74 (0.93–3.27) |
|  | 1 vs 3 | 0.2124 | 0.1908 | 619 | 1.11 | 0.2661 | –0.1623 | 0.5871 | 1.24 (0.85–1.80) |
|  | 2 vs 3 | -0.3434 | 0.2780 | 619 | -1.24 | 0.2172 | –0.8892 | 0.2025 | 0.71 (0.41–1.22) |
|  | 1 vs 4 | 0.7237 | 0.2254 | 619 | 3.21 | 0.0014 | 0.2810 | 1.1664 | 2.06 (1.32–3.21) |
|  | 2 vs 4 | 0.1680 | 0.3009 | 619 | 0.56 | 0.5769 | –0.4230 | 0.7590 | 1.18 (0.66–2.14) |
| Anxiety | 1 TxCYC | -0.3269 | 0.1637 | 618 | -2.00 | 0.0463 | –0.6484 | -0.00537 | 0.72 (0.52–0.99) |
|  | 2 TxHIK | -0.3635 | 0.2330 | 618 | -1.56 | 0.1192 | –0.8211 | 0.09399 | 0.70 (0.44–1.10) |
|  | 3 TxCON | -0.1445 | 0.07320 | 618 | -1.97 | 0.0488 | –0.2883 | -0.00075 | 0.87 (0.75–0.99) |
|  | 4 HCON | -0.7994 | 0.1524 | 618 | -5.24 | <.0001 | –1.0988 | -0.5001 | 0.45 (0.33–0.61) |
|  | 1 vs 2 | 0.0367 | 0.2845 | 618 | 0.13 | 0.8975 | –0.5220 | 0.5953 | 1.04 (0.59–1.81) |
|  | 1 vs 3 | -0.1824 | 0.1791 | 618 | -1.02 | 0.3089 | –0.5340 | 0.1693 | 0.83 (0.59–1.18) |
|  | 2 vs 3 | -0.2190 | 0.2440 | 618 | -0.90 | 0.3696 | –0.6981 | 0.2600 | 0.80 (0.50–1.30) |
|  | 1 vs 4 | 0.4726 | 0.2227 | 618 | 2.12 | 0.0342 | 0.03531 | 0.9098 | 1.60 (1.04–2.48) |
|  | 2 vs 4 | 0.4359 | 0.2774 | 618 | 1.57 | 0.1166 | –0.1089 | 0.9807 | 1.55 (0.90–2.67) |

* 1: cycling transplant recipients; 2: hiking transplant recipients; 3: control transplant recipients; 4: healthy participants.
