# Supplementary material for: Patient-reported outcomes evaluation and assessment of facilitators and barriers to physical activity in the Transplantoux aerobic exercise intervention
Source: PLoS One. 2022 Oct 26;17(10):e0273497. doi: 10.1371/journal.pone.0273497 (PMC9605336; doi:10.1371/journal.pone.0273497)
Supplement: S4 Table — * 1: cycling transplant recipients; 2: hiking transplant recipients; 3: control transplant recipients; 4: healthy participants. (DOCX) [file pone.0273497.s005.docx]

**Supporting information:**

Table S4: Regression slopes per study group and slope contrasts of patient-reported outcomes over time between intervention (T3, month 6) and study end (T5, month 12)

| General mixed models | | |  |  |  |  |  |  | | |  | |  | |  |
| --- | --- | --- | --- | --- | --- | --- | --- | --- | --- | --- | --- | --- | --- | --- | --- |
|  | Variable | Group | Estimate | SE | DF | t Value | Pr > \|t\| | Lower | | | Upper | | OR (95%CI) | |  |
| Physical activity: MET-min per week (square root) | | 1 TxCYC | -3.6591 | 2.1558 | 426 | -1.70 | 0.0904 | | -7.8964 | 0.5781 | | | / | |  |
|  |  | 2 TxHIK | 1.1379 | 3.4353 | 426 | 0.33 | 0.7406 | | -5.6145 | 7.8902 | | | / | |  |
|  |  | 3 TxCON | -2.3996 | 1.1743 | 426 | -2.04 | 0.0416 | | -4.7078 | -0.09136 | | | / | |  |
|  |  | 4 HCON | -5.3145 | 1.8792 | 426 | -2.83 | 0.0049 | | -9.0083 | -1.6208 | | | / | |  |
|  |  | 1 vs 2 | -4.7970 | 4.0557 | 426 | -1.18 | 0.2376 | | -12.7687 | 3.1747 | | | / | |  |
|  |  | 1 vs 3 | -1.2596 | 2.4549 | 426 | -0.51 | 0.6082 | | -6.0848 | 3.5656 | | | / | |  |
|  |  | 2 vs 3 | 3.5374 | 3.6305 | 426 | 0.97 | 0.3304 | | -3.5985 | 10.6734 | | | / | |  |
|  |  | 1 vs 4 | 1.6554 | 2.8599 | 426 | 0.58 | 0.5630 | | -3.9658 | 7.2766 | | | / | |  |
|  |  | 2 vs 4 | 6.4524 | 3.9157 | 426 | 1.65 | 0.1001 | | -1.2442 | 14.1490 | | | / | |  |
| SF-36 physical component score (squared) | | 1 TxCYC | -53.7602 | 131.83 | 470 | -0.41 | 0.6836 | | -312.81 | 205.29 | | | / | |  |
|  |  | 2 TxHIK | -268.89 | 208.25 | 470 | -1.29 | 0.1973 | | -678.10 | 140.33 | | | / | |  |
|  |  | 3 TxCON | -4.3383 | 69.2418 | 470 | -0.06 | 0.9501 | | -140.40 | 131.72 | | | / | |  |
|  |  | 4 HCON | -398.22 | 111.34 | 470 | -3.58 | 0.0004 | | -617.01 | -179.43 | | | / | |  |
|  |  | 1 vs 2 | 215.13 | 246.47 | 470 | 0.87 | 0.3832 | | -269.19 | 699.45 | | | / | |  |
|  |  | 1 vs 3 | -49.4219 | 148.91 | 470 | -0.33 | 0.7401 | | -342.03 | 243.19 | | | / | |  |
|  |  | 2 vs 3 | -264.55 | 219.46 | 470 | -1.21 | 0.2286 | | -695.79 | 166.69 | | | / | |  |
|  |  | 1 vs 4 | 344.46 | 172.56 | 470 | 2.00 | 0.0465 | | 5.3786 | 683.55 | | | / | |  |
|  |  | 2 vs 4 | 129.33 | 236.15 | 470 | 0.55 | 0.5842 | | -334.70 | 593.37 | | | / | |  |
| SF-36 mental component score (squared) | | 1 TxCYC | 152.10 | 139.32 | 470 | 1.09 | 0.2755 | | -121.66 | 425.86 | | | / | |  |
|  |  | 2 TxHIK | -77.9416 | 220.13 | 470 | -0.35 | 0.7234 | | -510.49 | 354.61 | | | / | |  |
|  |  | 3 TxCON | -79.4996 | 73.1700 | 470 | -1.09 | 0.2778 | | -223.28 | 64.2813 | | | / | |  |
|  |  | 4 HCON | -544.78 | 117.59 | 470 | -4.63 | <.0001 | | -775.84 | -313.72 | | | / | |  |
|  |  | 1 vs 2 | 230.04 | 260.51 | 470 | 0.88 | 0.3777 | | -281.87 | 741.94 | | | / | |  |
|  |  | 1 vs 3 | 231.60 | 157.36 | 470 | 1.47 | 0.1418 | | -77.6252 | 540.82 | | | / | |  |
|  |  | 2 vs 3 | 1.5580 | 231.97 | 470 | 0.01 | 0.9946 | | -454.26 | 457.38 | | | / | |  |
|  |  | 1 vs 4 | 696.88 | 182.31 | 470 | 3.82 | 0.0001 | | 338.64 | 1055.12 | | | / | |  |
|  |  | 2 vs 4 | 466.84 | 249.56 | 470 | 1.87 | 0.0620 | | -23.5546 | 957.24 | | | / | |  |
| Euroqol VAS (squared) | | 1 TxCYC | -199.39 | 128.71 | 472 | -1.55 | 0.1220 | | -452.30 | 53.5195 | | | / | |  |
|  |  | 2 TxHIK | 111.01 | 203.37 | 472 | 0.55 | 0.5854 | | -288.63 | 510.64 | | | / | |  |
|  |  | 3 TxCON | -154.89 | 67.4512 | 472 | -2.30 | 0.0221 | | -287.43 | -22.3512 | | | / | |  |
|  |  | 4 HCON | -262.11 | 108.61 | 472 | -2.41 | 0.0162 | | -475.52 | -48.6996 | | | / | |  |
|  |  | 1 vs 2 | -310.40 | 240.68 | 472 | -1.29 | 0.1978 | | -783.33 | 162.54 | | | / | |  |
|  |  | 1 vs 3 | -44.4965 | 145.31 | 472 | -0.31 | 0.7596 | | -330.03 | 241.04 | | | / | |  |
|  |  | 2 vs 3 | 265.90 | 214.27 | 472 | 1.24 | 0.2152 | | -155.14 | 686.94 | | | / | |  |
|  |  | 1 vs 4 | 62.7220 | 168.41 | 472 | 0.37 | 0.7097 | | -268.20 | 393.64 | | | / | |  |
|  |  | 2 vs 4 | 373.12 | 230.56 | 472 | 1.62 | 0.1063 | | -79.9279 | 826.16 | | | / | |  |
| Generalized mixed models | | |  |  |  |  |  |  | | | |  | |  | |
|  | Variable | Group | Estimate | SE | DF | t Value | Pr > \|t\| | Lower | | | Upper | | OR (95%CI) | |  |
| Physical activity: Status | | 1 TxCYC | -0.5236 | 0.2999 | 467 | -1.75 | 0.0815 | 0.0658 | | | | -1.1130 | | 0.59 (0.33-1.07) | |
|  |  | 2 TxHIK | 0.5136 | 0.4840 | 467 | 1.06 | 0.2891 | 1.4647 | | | | -0.4374 | | 1.67 (0.65-4.33) | |
|  |  | 3 TxCON | -0.1062 | 0.1408 | 467 | -0.75 | 0.4511 | 0.1705 | | | | -0.3828 | | 0.90 (0.68-1.19) | |
|  |  | 4 HCON | -0.6198 | 0.2447 | 467 | -2.53 | 0.0116 | -0.1391 | | | | -1.1006 | | 0.54 (0.33-0.87) | |
|  |  | 1 vs 2 | -1.0373 | 0.5705 | 467 | -1.82 | 0.0697 | 0.0838 | | | | -2.1583 | | 0.35 (0.16-1.08) | |
|  |  | 1 vs 3 | -0.4174 | 0.3305 | 467 | -1.26 | 0.2071 | 0.2319 | | | | -1.0668 | | 0.66 (0.34-1.26) | |
|  |  | 2 vs 3 | 0.6198 | 0.5045 | 467 | 1.23 | 0.2198 | 1.6111 | | | | -0.3715 | | 1.86 (0.69-5.01) | |
|  |  | 1 vs 4 | 0.0962 | 0.3846 | 467 | 0.25 | 0.8026 | 0.8520 | | | | -0.6596 | | 1.10 (0.52-2.34) | |
|  |  | 2 vs 4 | 1.1335 | 0.5436 | 467 | 2.09 | 0.0376 | 2.2016 | | | | 0.0653 | | 3.10 (1.07-9.04) | |
| Mental health | | 1 TxCYC | -0.3472 | 0.3360 | 461 | -1.03 | 0.3020 | -1.0076 | | | | 0.3131 | | 0.71 (0.37–1.37) | |
|  |  | 2 TxHIK | 0.3937 | 0.5984 | 461 | 0.66 | 0.5110 | -0.7823 | | | | 1.5697 | | 1.48 (0.46–4.80) | |
|  |  | 3 TxCON | 0.1955 | 0.1396 | 461 | 1.40 | 0.1618 | -0.0786 | | | | 0.4698 | | 1.22 (0.92–1.60) | |
|  |  | 4 HCON | 0.9899 | 0.2791 | 461 | 3.55 | 0.0004 | 0.4415 | | | | 1.5383 | | 2.69 (1.55–4.66) | |
|  |  | 1 vs 2 | -0.7409 | 0.6870 | 461 | -1.08 | 0.2814 | -2.0909 | | | 0.6091 | | 0.48 (0.12–1.84) | |  |
|  |  | 1 vs 3 | -0.5428 | 0.3645 | 461 | -1.49 | 0.1371 | -1.2590 | | | 0.1734 | | 0.58 (0.28–1.19) | |  |
|  |  | 2 vs 3 | 0.1981 | 0.6141 | 461 | 0.32 | 0.7471 | -1.0086 | | | 1.4049 | | 1.22 (0.36–4.08) | |  |
|  |  | 1 vs 4 | -1.3371 | 0.4388 | 461 | -3.05 | 0.0024 | -2.1993 | | | -0.4749 | | 0.26 (0.11–0.62) | |  |
|  |  | 2 vs 4 | -0.5962 | 0.6582 | 461 | -0.91 | 0.3655 | -1.8897 | | | 0.6973 | | 0.55 (0.15–2.01) | |  |
| Stress | | 1 TxCYC | 0.02958 | 0.2430 | 192 | 0.12 | 0.9033 | -0.4498 | | | | 0.5089 | | 1.03 (0.64–1.66) | |
|  |  | 2 TxHIK | 0.8821 | 0.3581 | 192 | 2.46 | 0.0146 | 0.1759 | | | | 1.5884 | | 2.42 (1.19–4.90) | |
|  |  | 3 TxCON | 0.1467 | 0.1251 | 192 | 1.17 | 0.2427 | -0.1002 | | | | 0.3935 | | 1.16 (0.90–1.48) | |
|  |  | 4 HCON | 0.8554 | 0.2308 | 192 | 3.71 | 0.0003 | 0.4001 | | | | 1.3106 | | 2.35 (1.49–3.70) | |
|  |  | 1 vs 2 | -0.8526 | 0.4327 | 192 | -1.97 | 0.0502 | -1.7059 | | | 0.00083 | | 0.43 (0.19–1.00) | |  |
|  |  | 1 vs 3 | -0.1171 | 0.2734 | 192 | -0.43 | 0.6690 | -0.6563 | | | 0.4222 | | 0.89 (0.52–1.53) | |  |
|  |  | 2 vs 3 | 0.7355 | 0.3788 | 192 | 1.94 | 0.0536 | -0.01160 | | | 1.4826 | | 2.09 (0.99–4.40) | |  |
|  |  | 1 vs 4 | -0.8258 | 0.3351 | 192 | -2.46 | 0.0146 | -1.4867 | | | -0.1649 | | 0.44 (0.23–0.85) | |  |
|  |  | 2 vs 4 | 0.02677 | 0.4231 | 192 | 0.06 | 0.9496 | -0.8078 | | | 0.8613 | | 1.03 (0.45–2.37) | |  |
| Depressive symptoms | | 1 TxCYC | -0.1302 | 0.2937 | 470 | -0.44 | 0.6577 | -0.7074 | | | | 0.4469 | | 0.88 (0.49–1.56) | |
|  |  | 2 TxHIK | 0.7291 | 0.4434 | 470 | 1.64 | 0.1007 | -0.1421 | | | | 1.6003 | | 2.07 (0.87–4.95) | |
|  |  | 3 TxCON | 0.3227 | 0.1380 | 470 | 2.34 | 0.0198 | 0.05147 | | | | 0.5940 | | 1.38 (1.05–1.81) | |
|  |  | 4 HCON | 0.8159 | 0.2465 | 470 | 3.31 | 0.0010 | 0.3316 | | | | 1.3003 | | 2.26 (1.39–3.67) | |
|  |  | 1 vs 2 | -0.8594 | 0.5326 | 470 | -1.61 | 0.1073 | -1.9060 | | | 0.1873 | | 0.42 (0.15–1.21) | |  |
|  |  | 1 vs 3 | -0.4530 | 0.3252 | 470 | -1.39 | 0.1643 | -1.0919 | | | 0.1860 | | 0.64 (0.33–1.20) | |  |
|  |  | 2 vs 3 | 0.4064 | 0.4629 | 470 | 0.88 | 0.3804 | -0.5032 | | | 1.3160 | | 1.50 (0.60–3.73) | |  |
|  |  | 1 vs 4 | -0.9462 | 0.3847 | 470 | -2.46 | 0.0143 | -1.7022 | | | -0.1902 | | 0.39 (0.18–0.83) | |  |
|  |  | 2 vs 4 | -0.08680 | 0.5040 | 470 | -0.17 | 0.8633 | -1.0771 | | | 0.9035 | | 0.92 (0.34–2.47) | |  |
| Anxiety | | 1 TxCYC | -0.05591 | 0.2769 | 469 | -0.20 | 0.8401 | -0.6000 | | | | 0.4882 | | 0.95 (0.55–1.63) | |
|  |  | 2 TxHIK | 0.4888 | 0.3573 | 469 | 1.37 | 0.1720 | -0.2133 | | | | 1.1909 | | 1.63 (0.81–3.29) | |
|  |  | 3 TxCON | 0.04785 | 0.1322 | 469 | 0.36 | 0.7175 | -0.2119 | | | | 0.3075 | | 1.05 (0.81–1.36) | |
|  |  | 4 HCON | 0.8925 | 0.2559 | 469 | 3.49 | 0.0005 | 0.3897 | | | | 1.3953 | | 2.44 (1.48–4.04) | |
|  |  | 1 vs 2 | -0.5447 | 0.4521 | 469 | -1.20 | 0.2289 | -1.4331 | | | | 0.3438 | | 0.58 (0.24–1.41) | |
|  |  | 1 vs 3 | -0.1038 | 0.3069 | 469 | -0.34 | 0.7354 | -0.7067 | | | | 0.4992 | | 0.90 (0.49–1.65) | |
|  |  | 2 vs 3 | 0.4409 | 0.3809 | 469 | 1.16 | 0.2476 | -0.3075 | | | | 1.1893 | | 1.55 (0.74–3.28) | |
|  |  | 1 vs 4 | -0.9484 | 0.3773 | 469 | -2.51 | 0.0123 | -1.6899 | | | | -0.2069 | | 0.39 (0.18–0.81) | |
|  |  | 2 vs 4 | -0.4038 | 0.4379 | 469 | -0.92 | 0.3570 | -1.2643 | | | | 0.4567 | | 0.67 (0.28–1.58) | |

* 1: cycling transplant recipients; 2: hiking transplant recipients; 3: control transplant recipients; 4: healthy participants.
