# Supplementary material for: Patient-reported outcomes evaluation and assessment of facilitators and barriers to physical activity in the Transplantoux aerobic exercise intervention
Source: PLoS One. 2022 Oct 26;17(10):e0273497. doi: 10.1371/journal.pone.0273497 (PMC9605336; doi:10.1371/journal.pone.0273497)
Supplement: S1 Protocol — (PDF) [file pone.0273497.s008.pdf]

Transplantoux Health Evaluation Study (THES):

Effect of an exercise training intervention on health-related quality of life and psychosocial and behavioral variables in adult solid organ transplant recipients: a quasi-experimental study design

Name of the applicants:

Evi Masschelein (Ph.D.)
Abdominale Transplantatieheelkunde
UZ Herestraat 49 - bus 7003 47
3000 Leuven
tel. +32 16 37 26 22
evi.masschelein@kuleuven.be

Laurens J Ceulemans (M.D.)
Abdominale Transplantatieheelkunde
UZ Herestraat 49 - bus 7003 47
3000 Leuven
tel. +32 16 34 87 27
laurens.ceulemans@uzleuven.be

Sabina De Geest (R.N., Ph.D.)
Centrum voor Ziekenhuis- en Verplegingswetenschap
Kapucijnenvoer 35 blok d - bus 7001
3000 Leuven
tel. +32 16 37 32 94
fax +32 16 3 36970
Institute of Nursing Science, University of Basel (CH)
sabina.degeest@unibas.ch

Diethard Monbaliu (M.D., Ph.D.)
Abdominale Transplantatieheelkunde
UZ Herestraat 49 - bus 7003 47
3000 Leuven
tel. +32 16 3 48727
fax +32 16 3 48743
diethard.monbaliu@uzleuven.be

BACKGROUND

During the last decades advances in solid organ transplantation, like e.g. organ preservation, surgical techniques and immunosuppressive treatment have contributed to improvements in postoperative survival. Thereby, solid organ transplantation (Tx) has moved from experimental treatment towards a clinical reality and a life-saving surgery, offering most patients a good long-term survival. However, further improvement in long-term survival remains a major challenge. Most improvements in survival have indeed primarily been achieved through improvement in 1st year survival. It is increasingly recognized that non-pharmacological interventions hold the potential to improve long-term outcomes in transplantation as evidence shows that health behavior has been independently associated with transplant outcome (e.g. physical activity, non-smoking, medication adherence). Furthermore, exercise training interventions show positive outcome in a number of populations and limited research in transplantation shows positive outcomes in view of exercise capacity [1–8], muscle strength [5,7,9], cardiopulmonary variables [1,7,9] and body composition [8,10,11]. Finally there is a lack of research that evaluates exercise training interventions in view of patient reported outcomes (PROs) in transplant recipients.

PROs are likely to become more important in future besides any other clinical or physiological outcomes because information is collected directly from the patient without interpretation by clinicians or others[12]. The most common PRO assessed in a patient population is health-related quality-of-life (HRQOL). For example, the Medical Outcomes Study Short Form questionnaire (SF-36) has been used in many previous studies in transplantation and demonstrated a high reliability and validity for assessment of the global self-reported HRQOL[13].

Previous research in transplant recipients shows only a small number of good-quality studies evaluating the effect of exercise training interventions on HRQOL. Two studies in heart transplant recipients [4,14], two studies in lung transplant recipients [6,7] and one study in liver transplant recipients [8] showed improved HRQOL after exercise training. Above studies are randomized controlled trials, however sample size was small. Furthermore, most research is dedicated to single-organ research. Although several RCTs about exercise training interventions and HRQOL have been conducted in transplant recipients, questions still remain about the long-term effects of exercise training on sustained improvements in HRQOL. To our knowledge, few good-quality research is available about exercise training and psychosocial and behavioral variables in transplant recipients.

Moreover, the majority of exercise training studies have been focused on heart and lung transplant recipients[15]. However, limited exercise capacity pre- and posttransplant is seen in all organ transplants, therefore, more research about exercise training studies in liver and kidney transplant recipients is also needed. Given that physical activity / exercise training is a relevant non-pharmacological intervention in all solid organ transplant recipients and given the positive effects in transplant and non-transplant populations of physical activity / exercise training programs, testing of the effect of physical activity interventions can be broadened from single organ transplant groups to a group including different types of organ transplants.

To date, there is a lack of knowledge why transplant recipients do not engage in regular exercise. To our knowledge only one study in kidney transplant recipients studied the perceived barriers and motivators to exercise[16]. However understanding the barriers and motivators will help to tailor exercise interventions and increase the physical activity level in transplant recipients.

In the University Hospitals of Leuven (Belgium) a project named 'Transplantoux' has been launched to enhance physical activity and exercise capacity in transplant recipients who had been successfully

transplanted for heart, lung, liver, kidney or small bowel failure[17]. Transplant recipients participate in the 'Transplantoux' exercise training intervention for 6-months with the ultimate goal to cycle or hike up the Mont Ventoux (France) (distance: 25.9 km, mean slope: 4.4%). Extending on a previous safety feasibility study in which we demonstrated that selected Transplant recipients can safely participate in an intense exercise program and that exercise capacity was significantly improved after training [18,19]. The primary aim of the current study is to evaluate the short- and long-term effect of the 'Transplantoux' intervention in view of selected Patient Reported Outcomes (PRO), i.e. HRQOL, psychosocial and behavioral variables, using a quasi-experimental design with matched control sample of transplant recipients not participating in Transplantoux.

OBJECTIVES & AIMS

1. Primary aim:
   1.1. To compare the effect of Transplantoux exercise training intervention in solid organ transplant recipients in view of selected PROs from baseline until 6-months after end intervention, with matched control transplant recipients (i.e. age, gender, height, weight, type of transplantation and time since transplantation) (quasi-experimental design)

2. Secondary aim:
   2.1. To compare the effect of Transplantoux exercise training intervention in solid organ transplant recipients with the effect of Transplantoux exercise training intervention in healthy controls in view of selected PRO from baseline until 6-months follow-up (quasi-experimental design).
   2.2. To compare baseline characteristics of the three groups with matched controls from a representative sample of the Belgian population (cross sectional design 1 – beginning data collection)
   2.3. To compare perceived barriers and motivators for physical exercise/activity in solid organ transplant recipients participating in TRANSPLANTOUX with solid organ transplant recipients not participating in TRANSPLANTOUX (matched control sample) and with healthy controls participating in TRANSPLANTOUX (cross sectional design 2 – end data collection)

METHODS

RESEARCH DESIGN

This study uses a hybrid design combining a quasi-experimental study design (aims 1.1 & 2.1); with 2 cross-sectional designs (aims 2.2 & 2.3). The quasi-experimental design comprises three non-randomized groups (Figure 1): 1) Transplant patients participating in Transplantoux (TP), 2) Matched transplant recipients control group not participating in TP; and 3) A healthy control group participating in TP.

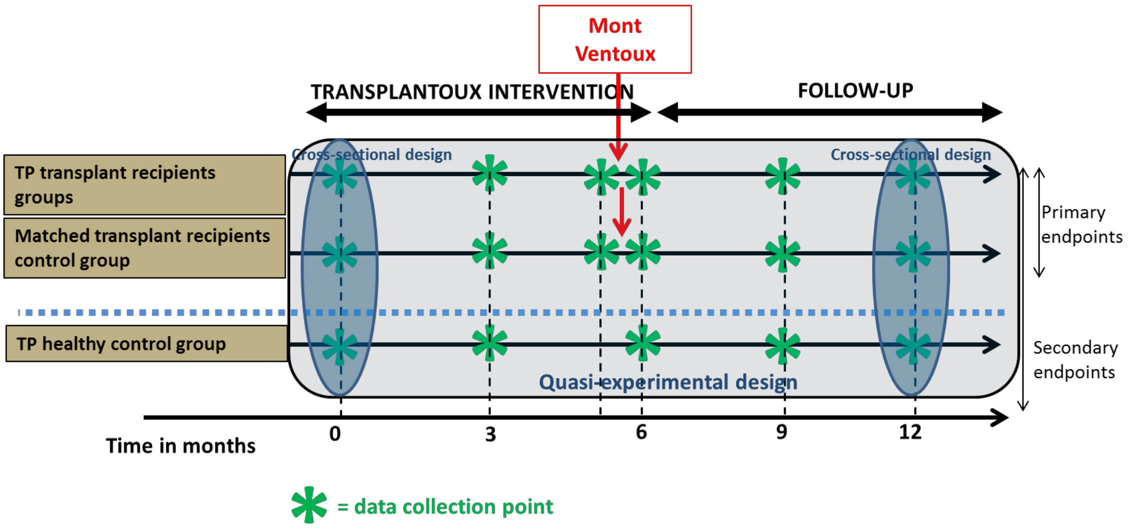

Figure 1. Hybrid design: Quasi-experimental study design & 2 cross sectional designs

SAMPLING

1) <u>TP transplant recipients group:</u>
Convenience sample of solid organ transplant recipients (heart, kidney, lung, liver, pancreas,small bowel) participating in TP.

- Inclusion Criteria:
  - Age between 18 – 70 years
  - More than 1y post-transplant
  - Written informed consent
- Exclusion Criteria:
  - Rejection last 6 months
  - Severe co-morbidity
  - Contra-indication for severe exercise (e.g. cardiovascular disease)
  - Insufficient knowledge of Dutch language

2) <u>Matched transplant recipients control group:</u>
Controls are retrieved from the Leuven University Hospital heart, kidney, lung, liver, pancreas, small bowel transplant database.

- Inclusion Criteria:
  - Age between 18 – 70 years

- More than 1y post-transplant
- Written informed consent
- Exclusion Criteria:
    - Rejection last 6 months
    - Insufficient knowledge of Dutch language

See inferential statistics matching below.

3) TP healthy control group:
Convenience sample of health care providers (e.g. doctor, paramedic or medical companion involved in care programs for organ Tx) and TP patient's family members and friends participating in Transplantoux.

- Inclusion Criteria:
    - Age between 18 – 70 years
    - Informed consent
- Exclusion Criteria:
    - Contra-indication for severe exercise (e.g. cardiovascular disease) (through medical doctor at home)
    - Insufficient knowledge of Dutch language

TRANSPLANTOUX INTERVENTION

1) TP transplant recipients group:
The Transplantoux intervention consists of a 6-month independent home-based exercise training program (cycling/hiking) combined with 7 supervised group training sessions and at the end of the training program the climb of the Mont Ventoux.

Home-based personalized exercise training program (cycling/hiking)
At the start of the study transplant recipients undergo a cardiopulmonary exercise test (CPET) on a cycle ergometer. Based on these results, an individualized training program is prescribed by a physical therapist. The training program consists of *cycling* or *hiking* training sessions dependent on the choice of the transplant recipient. The prescription includes 6-months independent home-based cardiovascular exercise. Patients exercise for at least three times per week with a minimal training duration of 30 minutes phases. In the first phase (month 1-2) the purpose is to build a higher exercise capacity and subjects cycle/walk volumes at low intensity (60% of maximal heart rate).In the second phase (month 3-4) the intensity of training is slightly increased (70% of maximal heart rate). In the last phase (months 5-6) the volume of training is maintained and focused on intensive interval training (80% of maximal heart rate). Patients are contacted on monthly basis by e-mail to assess progress and adherence to the program.

Supervised group training sessions (cycling/hiking)
The supervised group training sessions by physiotherapists start in month 3. Every two weeks transplant recipients participate in a supervised group training session (in total 7 sessions). The distance of the cycling rides and hiking tours vary from 40 km to 135 km and 5 km to 20 km, respectively, depending on the individual level of the transplant recipient.

Climb of the Mont Ventoux (cycling/hiking)
The ultimate goal of the training intervention is to cycle or hike up the Mont Ventoux for 25.7 km with an average gradient slope of 4.5%.

TP healthy control group:

No home-based personalized exercise training program & no CPET

Supervised group training sessions (cycling/hiking)
The supervised group training sessions start in month 3. Every two weeks the healthy control group participate together with the TP transplant recipient group in a supervised group training session (in total 7 sessions). The distance of the cycling rides and hiking tours vary from 40 km to 135 km and 5 km to 20 km, respectively, depending on the individual level of the control person.

Climb of the Mont Ventoux (cycling/hiking)
The ultimate goal of the training intervention is to cycle or hike up the Mont Ventoux for 25.7 km with an average gradient slope of 4.5%.

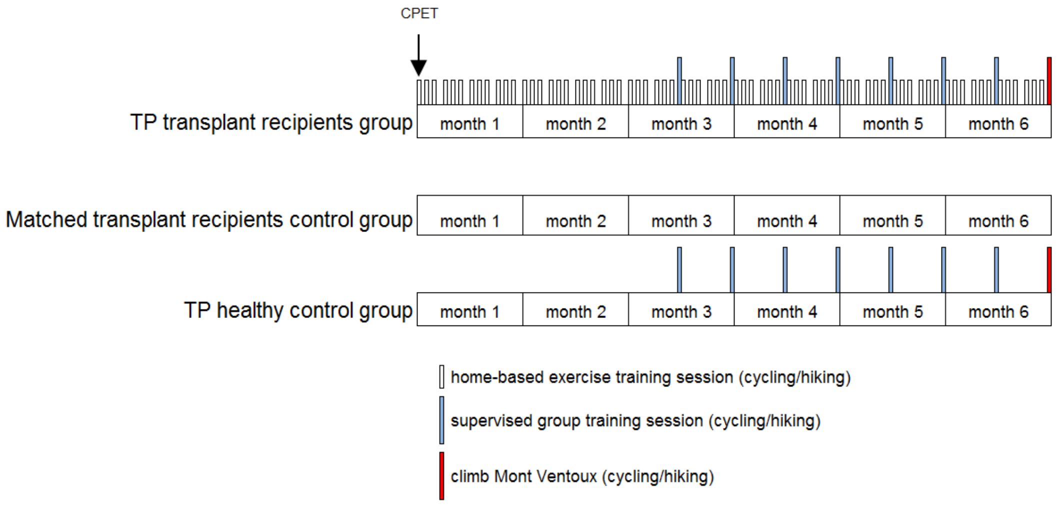

Figure 2. Transplantoux intervention. Cardiopulmonary exercise test (CPET)

VARIABLES AND MEASUREMENT

Demographic variables

- Basic demographic data for TP transplant recipients and matched control transplant recipients (i.e. age, gender, height, weight, type of transplantation and time since transplantation) is retrieved from medical files in the University Hospital of Leuven.
- Basic demographic data for the healthy control group (i.e. age, gender, height and weight) is retrieved from a self-report questionnaire.

Socioeconomic variables
Socio-economic factors (i.e. educational, occupational, and civil status) are retrieved from the self-report questionnaire.

Patient Reported outcomes
Selected PROs were derived from established instruments or the Belgian Health Survey.

- Perceived quality of life:

- SF-36, Self-reported domains of health status (36 items, 8 subscales, 2-point/3-point/5-point/6-point Likert-scale)[20]:

  The SF-36 is a 36- item questionnaire that includes eight components of health-related quality of life: physical functioning (PF), role limitations owing to physical health (RP), body pain (BP), general health (GH), vitality (VT), social functioning (SF), role limitations due to emotional health (RE) and mental health (MH). These scales result in a score from 0 to 100, with higher scores being more positive (i.e. less pain, less limitation). The physical scale summary (PCS) is scored using weighted scores from all scales with higher weights from the PF, RP, BP and GH scales. The mental summary scale (MCS), likewise is scored using weighted scores from all individual scales with higher weights from the VT, SF, RE and MH scales.
- Belgian Health Survey 2013
  - EQ-5D, European Quality of Life-5 Dimensions (5 items, 5-point Likert scale)

    Mobility, self-care, usual activities, pain/discomfort and anxiety/depression
  - EQ-VAS, European Quality of Life-visual analogue scale (1 item, scoring 0-100) -

<u>Depressive symptomatology:</u>

- DASS-21, Depression, Anxiety and Stress Scale (21 items; 3 subscales; 4-pointL i k e r t -scale)[21]

  DASS-21 is a short form of DASS which is a self-report 4-point Likert scale and composed of three subscales: Depression (DASS-D), Anxiety (DASS-A), and Stress (DASS-S). The DASS-21 measures each of the three mental health conditions, over the past week, through seven items. Responses on each item range was from 0 (did not apply to me at all) to 3 (applied to me very much). The intensity of any of the three conditions is determined by the sum scores of responses to its 7-item subscale. T h e resulting score can be interpreted as follows: 0–4: no depressive symptomatology; 5– 6: mild; 7–10: moderate; 11–13: severe; and ≥14: extremely severe.

<u>- Stress and well-being:</u>

- Belgian Health Survey, 2013: GHQ-12, General Health Questionnaire (12 items, scoring method bimodal 0-0-1-1)[22]

  The GHQ is a screening tool which is used to identify the severity of psychological distress experienced by an individual within the past few weeks. Each item on the scale has four responses from "better than usual" to "much less than usual". The scores are summed up by adding all the items on the scale ranging from 0 to 12. a score of 4 or more is used to identify a participant with high-stress level.

<u>- Physical activity:</u>

- Belgian Health Survey, 2013: IPAQ, International Physical Activity Questionnaire (6 items, 3 subscales; categorical and continuous scoring):

  The IPAQ short form asks about three specific types of activity. The specific types of activity that are assessed are walking, moderate-intensity activities and vigorous intensity activities; frequency (measured in days per week) and duration (time per day) are collected separately for each specific type of activity.

<u>- Social contacts and social support:</u>

- Belgian Health Survey, 2013 (5 items)

<u>- Barriers and motivators to exercise:</u>

- Barriers and Motivators questionnaire (55 items, 4-point Likert scale):

The barriers and motivators questionnaire is a self-reported questionnaire designed to identify barriers and motivators of physical activity in the hemodialysis population[23]. Two barriers score are derived from the questionnaire: the Barrier Frequency Score and Barrier Intensity Score. The Barrier Frequency score is obtained by summing all of the barriers endorsed at least slightly. The Barrier Intensity score is derived by assigning progressively higher number to the intensity at which a barrier was endorsed(i.e. not at all a barrier = 0, slightly a barrier = 1, moderately a barrier = 2, and very much a barrier = 3), and then summing these for each barrier across the sample response. Also for the motivators of physical activity, two motivators scores are derived: the Motivator Frequency Score and Motivator Intensity score.[23]

DATA COLLECTION

Time frame of data collection

Data collection will be performed at baseline, 3 months (in the middle of the training intervention), 6 months (immediately following the intervention), 9 months (3 months follow-up period) and 12 months (6 months follow-up period). TP transplant recipients group and TP healthy control group will have an additional measurement just before the climb of the Mont Ventoux (see Figure 1).

Data collection takes place from January 2015 until January 2016. Telephone numbers, email addresses and post addresses of all Transplantoux participants (transplant recipients & healthy controls) are retrieved from the organization Transplantoux. Telephone numbers, email addresses and post addresses of the matched transplant recipients control group are extracted from the University Hospital Leuven database. All subjects receive a short call about the purpose of the study. Thereafter, subjects receive an email with background information and an invitation to participate in the study with a link to the online survey. The online survey is completed via a webbased survey tool provided by KU Leuven (www.websurvey.kuleuven.be). All subjects sign an online informed consent. If subjects have no access to the internet, a survey is send by post and subjects have to send the completed survey back with a prepaid return envelope. Each survey has a unique code that allows identification of the subject by the investigator.

Subjects who not fill in the questionnaire after 2 weeks receive a reminder. If they do not return the questionnaire 7 days after the first reminder, a 2nd reminder is sending. If they still not return the questionnaire after the 2nd reminder, they receive a reminder telephone call (only at baseline measurement). If a subject does not complete the survey after the reminders, he is a non-responder.

Data analysis

Data analysis will be performed in collaboration with S. Fieuws (statistical department)

A. Descriptive statistics

Descriptive statistics will be calculated as appropriate based on measurement level and distribution for demographic and socio-economic variables, and for the PROs at each timepoint.

B. Missing value analysis and management

Baseline characteristics and baseline PROs will be compared between subjects without missing values (complete cases) and subjects who have at least at one timepoint a missing value.

In the longitudinal part of study some drop-out is likely to happen. In the comparison ofthe evolutions between groups (aim1) and in the comparison referring to the end of treatment (aim 2.3), a statistical approach will be used (cfr infra) which can handle the presence of missing values and which is valid under a missing at random assumption (MAR). This assumption holds if the

presence of missing values is not related i.e. not related to unobserved information (i.e. unmeasured subject characteristics or unobserved levels of the longitudinal measurement). Hence, as opposed to a complete case analysis, the conclusions will still be valid if the probability of drop- out is related to a covariate included in the model (e.g. age) or to observed levels of the outcome analyzed.

C. Matching
   a. Computerized matching of cases to controls using the greedy matching algorithm aiming for a fixed number of controls per case (1:4 matching). Purpose is to end up with at least 3 participating controls per case. Controls are matched to cases based on type of transplant, gender, age and time since transplant. The distance between a case and control is defined as the weighted sum (over the matching variables) of the absolute case-control differences, with the weight for the time since transplant being the double of the weight for age. For type of transplant and gender, only exact matches are allowed. The matching has been performed using the macro GMATCH, written for SAS software, version 9.2 of the SAS System for Windows. References: Bergstralh, EJ and Kosanke JL(1995). Computerized matching of controls. Section of Biostatistics, Technical Report 56. Mayo Foundation.
   b. Propensity score matching for Belgian Health Survey.

D. Inferential statistics

General linear mixed models are used to evaluate differences in evolution of PROs between groups from baseline until end of follow-up. An unstructured covariance matrix will be used for the repeated measures over time. A simplification of this matrix (i.e. a reduction of the number of used covariance parameters) will be considered based on the AIC criterion. A random intercept for each cluster (a case and controls) will be added to model the correlation between the members of the same cluster. For each PRO, a transformation will be applied when needed to obtain a more symmetric distribution of the model residuals. Least-squares means and confidence intervals will be given after backtransformation to the original scale. To handle potential dropout, also the comparison at the end treatment (aim 2.3) will be based on the same model using all longitudinal measurements.

As an alternative approach (sensitivity analysis), the same analysis will be performed restricted to the post-baseline values, but with baseline as a covariate.

Linear mixed models with a random intercept for each cluster will be used to compare (matched) groups at baseline (aim 2.2).

The level of significance for all comparisons will be set at $P < 0.05$.

1. Primary outcomes:
   a. To compare the effect of TRANSPLANTOUX in solid organ transplant recipients participating in TRANSPLANTOUX with solid organ transplant recipients not participating in TRANSPLANTOUX (matched control sample) in view of selected PRO from baseline until 6-months follow-up (quasi-experimental design).

2. Secondary outcomes:
   a. To compare the effect of TRANSPLANTOUX in solid organ transplant recipients participating in TRANSPLANTOUX compared to healthy

controls participating in TRANSPLANTOUX in view of selected PRO from baseline until 6-months follow-up. (quasi-experimental design)

b. To compare baseline characteristics of the three groups withmatched controls from a representative sample of the Belgian population (Cross sectional design 1 -beginning data collection)
c. To compare perceived barriers and motivators for physical exercise/activity in solid organ transplant recipients participating in TRANSPLANTOUX with solid organ transplant recipients not participating in TRANSPLANTOUX (matched control sample) and with healthy controls participating in TRANSPLANTOUX (Cross sectional design 2 - end data collection)

FUNDING

Government of Flanders

REFERENCES

1. Bernardi L, Radaelli A, Passino C, et al. Effects of physical training on cardiovascular control after heart transplantation. *Int. J. Cardiol.* 2007;118(3):356-362. doi:10.1016/ j.ijcard.2006.07.032.

2. Braith RW, Schofield RS, Hill JA, Casey DP, Pierce GL. Exercise Training Attenuates Progressive Decline in Brachial Artery Reactivity in Heart Transplant Recipients. *J. Hear. Lung Transplant.* 2008;27:52-59. doi:10.1016/j.healun.2007.09.032.

3. Haykowsky M, Taylor D, Kim D, Tymchak W. Exercise training improves aerobic capacity and skeletal muscle function in heart transplant recipients. *Am. J. Transplant* 2009;9(4):734-9. doi:10.1111/j.1600-6143.2008.02531.x.

4. Wu Y-T, Chien C-L, Chou N-K, Wang S-S, Lai J-S, Wu Y-W. Efficacy of a home-based exercise program for orthotopic heart transplant recipients. *Cardiology* 2008;111(2):87-93. doi:10.1159/000119695.

5. Painter PL, Hector L, Ray K, et al. A randomized trial of exercise training after renal transplantation. *Transplantation* 2002;74(1):42-8. Available at: http:// www.ncbi.nlm.nih.gov/pubmed/12134097.

6. Ihle F, Neurohr C, Huppmann P, et al. Effect of inpatient rehabilitation on quality of life and exercise capacity in long-term lung transplant survivors: a prospective, randomized study. *J. Heart Lung Transplant.* 2011;30(8):912-9. doi:10.1016/j.healun.2011.02.006.

7. Langer D, Burtin C, Schepers L, et al. Exercise training after lung transplantation improves participation in daily activity: a randomized controlled trial. *Am. J. Transplant* 2012;12(6):1584-92. doi:10.1111/j.1600-6143.2012.04000.x.

8. Krasnoff JB, Vintro a Q, Ascher NL, et al. A randomized trial of exercise and dietary counseling after liver transplantation. *Am. J. Transplant* 2006;6(8):1896-905. doi:10.1111/j.1600-6143.2006.01391.x.

9. Hermann TS, Dall CH, Christensen SB, Goetze JP, Prescott E, Gustafsson F. Effect of high intensity exercise on peak oxygen uptake and endothelial function in long-term heart transplant recipients. *Am. J. Transplant* 2011;11:536-541. doi:10.1111/j.1600-6143.2010.03403.x.

10. Tegtbur U, Pethig K, Machold H, Haverich A, Busse M. Functional endurance capacity and exercise training in long-term treatment after heart transplantation. *Cardiology* 2003;99:171-176. doi:10.1159/000071245.

11. Braith RW, Mills RM, Welsch MA, Keller JW, Pollock ML. Resistance exercise training restores bone mineral density in heart transplant recipients. *J Am Coll Cardiol* 1996;28:1471-1477. Available at: http://www.ncbi.nlm.nih.gov/entrez/query.fcgi?cmd=Retrieve&db=PubMed&dopt=Citation&list_uids=8917260.

12. Deshpande PR, Rajan S, Sudeepthi BL, Abdul Nazir CP. Patient-reported outcomes: A new era in clinical research. *Perspect. Clin. Res.* 2011;2:137-44. doi:10.4103/2229-3485.86879.

13. Pinson CW, Feurer ID, Payne JL, Wise PE, Shockley S, Speroff T. Health-Related Quality of Life After Different Types of Solid Organ Transplantation. *Ann. Surg.* 2000;232(4):597-607. doi:10.1097/00000658-200010000-00015.

14. Christensen SB, Dall CH, Prescott E, Pedersen SS, Gustafsson F. A high-intensity exercise program improves exercise capacity, self-perceived health, anxiety and depression in heart transplant recipients: a randomized, controlled trial. *J. Heart Lung Transplant.* 2012;31(1):106-7. doi:10.1016/j.healun.2011.10.014.

15. Didsbury M, McGee RG, Tong A, et al. Exercise training in solid organ transplant recipients: a systematic review and meta-analysis. *Transplantation* 2013;95(5):679-87. doi:10.1097/TP.0b013e31827a3d3e.

16. Sanchez Z V, Cashion AK, Cowan PA, Jacob SR, Wicks MN, Velasquez-Mieyer P. Perceived barriers and facilitators to physical activity in kidney transplant recipients. *Prog.Transplant.* 2007;17:324-331.

17. Transplantoux. No Title. 2010. Available at: www.transplantoux.be.

18. Cappelle M, Masschlein E, Van Remoortel H, et al. Intensive physical exercise after organ transplantation: a retrospective study in selected patients. In: *BTS Annual Meeting.*; 2014.

19. Van Remoortel H, Masschelein E, Troosters T, et al. ffect of an intenstive exercise program on daily physical activity in solid organ Recipients. In: *BTS Annual Meeting.*; 2014.

20. Ware JE, Snow KK, Kosinski M, Gandek B. *SF-36 Health Survey Manual and Interpretation Guide.*; 1993:1 v. (various pagings). Available at: http://books.google.com/books/about/SF_36_health_survey.html?id=WJsgAAAAMAAJ.

21. Lovibond SH, Lovibond PF. *Manual for the Depression Anxiety Stress Scales.*; 1995:42. doi:DOI: 10.1016/0005-7967(94)00075-U.

22. Goldberg D, Williams P. *A Users's Guide to the General Health Questionnaire*. (NFER-Nelson, ed.). Windsor, UK; 1988.

23. Goodman E, Ballou M. Perceived Barriers and Motivators to Exercise in Hemodialysis Patients. *Nephrol. Nurs. J.* 2004;31(1):23-29.
