# Supplementary material for: Patient-reported outcomes evaluation and assessment of facilitators and barriers to physical activity in the Transplantoux aerobic exercise intervention
Source: PLoS One. 2022 Oct 26;17(10):e0273497. doi: 10.1371/journal.pone.0273497 (PMC9605336; doi:10.1371/journal.pone.0273497)
Supplement: S1 Dataset — (DOCX) [file pone.0273497.s009.docx]

Patient level data

| **Category** | **Variable** | **Name** | **Values** |
| --- | --- | --- | --- |
| Study related | ID | Patient identification |  |
|  | GROUP | Study group | 1=TxCYC; 2=TxHIK; 3=TxCON; 4=HCON |
| Demographic | AGE | Age at inclusion | Years |
|  | SEX | Gender | 0=Male; 1=Female |
|  | MARITAL | Marital status | Single; Married/cohabiting; Divorced; Widow(er) |
|  | EDUC | Education | 1=Primary; 2=Secondary; 3=Vocational / bachelor; 4=Master / PhD |
|  | ORGAN | Primary transplant | 1=Heart; 2=Kidney; 3=Liver; 4=Lung; 5=Other |
|  | YSINCETX | Time since transplantation | Years |
|  | SMOK | Smoking status | 0=Never; 1=Current;2=Past |
|  | PACKYEARS | Numer of pack years | Years |
| Barriers | B01 | Lack of motivation | 1-4 |
|  | B02 | Being too fatigued | 1-4 |
|  | B03 | Unpleasant sensations associated with exercise | 1-4 |
|  | B04 | Shortness of breath | 1-4 |
|  | B05 | Fear of making health worse | 1-4 |
|  | B06 | Preferring to spend time doing other things | 1-4 |
|  | B07 | Self-conscious about appearance while exercising | 1-4 |
|  | B08 | Lack of interest | 1-4 |
|  | B09 | Fear of falling | 1-4 |
|  | B10 | Depressive symptoms | 1-4 |
|  | B11 | Fear of injury | 1-4 |
|  | B12 | Lack of knowledge of benefits of exercise | 1-4 |
|  | B13 | Physically demanding job | 1-4 |
|  | B14 | Low expectations by self to exercise | 1-4 |
|  | B15 | Low expectations by health care providers | 1-4 |
|  | B16 | No place to sit down while exercising outside | 1-4 |
|  | B17 | Fear of increased pain | 1-4 |
|  | B18 | Poor sidewalks | 1-4 |
|  | B19 | Anxiety | 1-4 |
|  | B20 | Co-morbid health problems | 1-4 |
|  | B21 | Lack of access to exercise facilities | 1-4 |
|  | B22 | Lack of time | 1-4 |
|  | B23 | Not wanting to become more fatigued | 1-4 |
|  | B24 | Concern that exercise will make too thirsty | 1-4 |
|  | B25 | Bad weather | 1-4 |
|  | B26 | Low expectations by family or friends | 1-4 |
|  | B27 | Lack of encouragement to exercise | 1-4 |
|  | B28 | Financial resources | 1-4 |
|  | B29 | Fear of crime during outdoor exercise | 1-4 |
|  | B30 | Lack of support from family or friends | 1-4 |
| Motivators | M01 | Wanting to feel better | 1-4 |
|  | M02 | Having less pain | 1-4 |
|  | M03 | Wanting increased health | 1-4 |
|  | M04 | Wanting to manage weight | 1-4 |
|  | M05 | Having a supportive exercise leader | 1-4 |
|  | M06 | Knowing the value of increased exercise | 1-4 |
|  | M07 | Enjoying how exercise feels | 1-4 |
|  | M08 | Feeling healthy | 1-4 |
|  | M09 | Belief in one’s ability to be physically active | 1-4 |
|  | M10 | Encouragement from health care providers | 1-4 |
|  | M11 | Encouragement from family or friends | 1-4 |
|  | M12 | High expectations from family or friends | 1-4 |
|  | M13 | Wanting increased strength | 1-4 |
|  | M14 | Receiving information on how to exercise | 1-4 |
|  | M15 | Wanting to have decreased pain | 1-4 |
|  | M16 | Exercising with others | 1-4 |
|  | M17 | High expectations from health care providers | 1-4 |
|  | M18 | Wanting decreased depression | 1-4 |
|  | M19 | Wanting enhanced physical mobility | 1-4 |
|  | M20 | Wanting increased energy | 1-4 |
|  | M21 | Wanting decreased anxiety | 1-4 |
|  | M22 | Attending exercise class | 1-4 |
|  | M23 | Having financial resources | 1-4 |

Longitudinal data

| **Category** | **Variable** | **Name** | **Values** |
| --- | --- | --- | --- |
| Study related | ID | Patient identification |  |
|  | GROUP | Study group | 1=TxCYC; 2=TxHIK; 3=TxCON; 4=HCON |
|  | TIME | Follow up time point | 1=T1-0m; 2=T2-3m; 4=T3-6m; 5=T4-9m; 6=T5-12m |
| SF-36 | PCS | Physical Component Score | 0-100 |
|  | MCS | Mental Component Score | 0-100 |
| DASS-21 | DEP | Depression | 0-21 |
|  | ANX | Anxiety | 0-21 |
|  | STRE | Stress | 0-21 |
|  | DEP_CAT | Depression categorized | 1-5 |
|  | ANX_CAT | Anxiety categorized | 1-5 |
|  | STRE_CAT | Stress categorized | 0-5 |
| GHQ | GHQ | General Health Questionnaire Method scale | 0-12 |
| IPAQ | METWEEK | MET-min/week | 0- |
|  | ACT_CAT | Level of Physical Activity | 1-3 |
| Health-related quality of life | EQ_VAS | EuroQoL Visual Analogue Scale | 0-100 |
