# Supplementary material for: Patient-reported outcomes evaluation and assessment of facilitators and barriers to physical activity in the Transplantoux aerobic exercise intervention
Source: PLoS One. 2022 Oct 26;17(10):e0273497. doi: 10.1371/journal.pone.0273497 (PMC9605336; doi:10.1371/journal.pone.0273497)
Supplement: Attachment — Submitted filename: renamed_55a5f.docx [file pone.0273497.s010.docx]

**COMMENTS – Manuscript PONE-D-21-28527**

Title: “Patient-reported outcomes evaluation of the MVT exercise intervention and assessment of facilitators and barriers to physical activity in transplant recipients”

**Important note: This review pertains only to ‘statistical aspects’ of the study and so** **‘clinical aspects’ [like medical importance,** **relevance of the study,** **‘clinical significance and implication(s)’ of the whole study, etc.] are to be evaluated [should be assessed] separately/independently. Further please note that any ‘statistical review’ is generally done under the assumption that (such) study specific methodological [as well as execution] issues are perfectly taken care of by the investigator(s). This review is not an exception to that and so does not cover clinical aspects {however, seldom comments are made only if those issues are intimately / scientifically related & intermingle with ‘statistical aspects’ of the study}. Agreed that ‘statistical methods’ are used as just tools here, however, they are vital part of methodology [and so should be given due importance].**

**COMMENTS:** What exactly you want to convey when you say [in ‘Abstract-Methods] “matched control transplant recipients (TxCON, n=213), and healthy MVT participants (HCON, n=91)?”. My question is ‘how ‘matched control’(s) were selected [why ‘n’s so different, why (n=3–4 matched TxCON to n=1 transplant recipient participating in MVT) (line 173) then]? If you wish/want to indicate something other than usually known meaning of the term ‘matched control’, please explain. Otherwise explain ‘how done/achieved by method used’ [line 154: Members of the TxCON group were matched using propensity score matching on 4 parameters, need be explained though briefly].

Note that any regression techniques (of course, including general/ generalized linear mixed regression analysis) are not originally developed for comparison between the groups [please refer to lines 342-343: General(ized) linear regression analysis was also used to perform post-hoc testing for possible baseline differences in outcome variables between the study groups]. Also note that though the measures/tools used are appropriate, most of them {example: the 36-item Short Form Health Survey (SF-36), the Depression, Anxiety and Stress Scale (DASS-21), General Health Questionnaire (GHQ–12), etc., etc.} yield data that are in [at the most] ‘ordinal’ level of measurement [and not in ratio level of measurement for sure {as the score two times higher does not indicate presence of that parameter/phenomenon as double (for example, a Visual Analogue Scales VAS score or say ‘depression’ score)}]. Then application of suitable non-parametric test(s) is/are indicated/advisable [even if distribution may be ‘Gaussian’ (i.e. normal)]. Agreed that there is/are no non-parametric test(s)/technique(s) available to be used as alternative in all situation(s) [suitable / most desired/applicable], but should be used whenever/wherever they are available.

Considering aims of the study, I wonder, if inclusion of fourth group [sample of healthy MVT participants (HCON)] was needed at all? Are the “conclusion(s)” [particularly line 90 : Transplantoux's MVT exercise intervention barely affected PROs] given in ‘Abstract’ and given at end [lines 586 onwards] same/on similar lines? What exactly you want convey by the term ’barely affected’? Remember that “Absence of evidence is not evidence of absence” [Altman DG, Bland JM. BMJ volume 311, 1995, p 485 (Reprinted : Australian Veterinary Journal 1996;74, 311)]. {Even when P-value is not significantly lower (unfortunately there is no direct comparison between groups – which is desirable in this case) that is null hypothesis of no difference is not rejected, (in short, result is not significant), that does not amount to evidence of absence i.e. it does imply that there no difference. It only implies that there is no (i.e. these samples do not provide) enough evidence to prove (rather indicate with certain specified confidence level) the difference}.

I do not have any specific recommendation.
